# Identification of Verrucarin A as a Potent and Selective Steroid Receptor Coactivator-3 Small Molecule Inhibitor

**DOI:** 10.1371/journal.pone.0095243

**Published:** 2014-04-17

**Authors:** Fei Yan, Yang Yu, Dar-Chone Chow, Timothy Palzkill, Franck Madoux, Peter Hodder, Peter Chase, Patrick R. Griffin, Bert W. O'Malley, David M. Lonard

**Affiliations:** 1 Department of Molecular and Cellular Biology, Baylor College of Medicine, Houston, Texas, United States of America; 2 Department of Pharmacology, Baylor College of Medicine, Houston, Texas, United States of America; 3 Department of Molecular Therapeutics, The Scripps Research Institute, Scripps Florida, Jupiter, Florida, United States of America; Florida International University, United States of America

## Abstract

Members of the steroid receptor coactivator (SRC) family are overexpressed in numerous types of cancers. In particular, steroid receptor coactivator 3 (SRC-3) has been recognized as a critical coactivator associated with tumor initiation, progression, recurrence, metastasis, and chemoresistance where it interacts with multiple nuclear receptors and other transcription factors to enhance their transcriptional activities and facilitate cross-talk between pathways that stimulate cancer progression. Because of its central role as an integrator of growth signaling pathways, development of small molecule inhibitors (SMIs) against SRCs have the potential to simultaneously disrupt multiple signal transduction networks and transcription factors involved in tumor progression. Here, high-throughput screening was performed to identify compounds able to inhibit the intrinsic transcriptional activities of the three members of the SRC family. Verrucarin A was identified as a SMI that can selectively promote the degradation of the SRC-3 protein, while affecting SRC-1 and SRC-2 to a lesser extent and having no impact on CARM-1 and p300 protein levels. Verrucarin A was cytotoxic toward multiple types of cancer cells at low nanomolar concentrations, but not toward normal liver cells. Moreover, verrucarin A was able to inhibit expression of the SRC-3 target genes MMP2 and MMP13 and attenuated cancer cell migration. We found that verrucarin A effectively sensitized cancer cells to treatment with other anti-cancer drugs. Binding studies revealed that verrucarin A does not bind directly to SRC-3, suggesting that it inhibits SRC-3 through its interaction with an upstream effector. In conclusion, unlike other SRC SMIs characterized by our laboratory that directly bind to SRCs, verrucarin A is a potent and selective SMI that blocks SRC-3 function through an indirect mechanism.

## Introduction

The p160 steroid receptor coactivator (SRC) family contains three members, SRC-1[1], SRC-2/GRIP1/TIF2 [2], [3] and SRC-3/Amplified in Breast Cancer-1 [4] that interact with multiple nuclear receptors (NRs) and other transcription factors to regulate gene transcription. The N-terminus of SRCs contains a conserved bHLH-PAS (basic Helix Loop Helix-Per Arnt Sims) motif [5] involved in protein-protein interactions [6]–[8]. The central region of SRCs contains the NR interaction domain (RID), including three α-helical LXXLL motifs for interaction with NRs [9], [10]. The C-terminal region of SRCs contains two activation domains (ADs), AD1 and AD2 that interact with other coactivators. AD1 interacts with p300/CBP while the AD2 binds to two histone methyltransferases - coactivator-associated arginine methyltransferase 1 (CARM1) and protein arginine methyltransferases (PRMT1) [11]–[14]. The C-terminal domain of SRC-1 and SRC-3 also contains weak HAT activity [15], [16].

All three SRCs have been implicated as oncoproteins. SRC-1 is overexpressed in a large subset of breast cancers and its overexpression is positively correlated with poor survival and knockdown of SRC-1 can inhibit breast cancer cell growth [17]. Other reports have implicated SRC-1 overexpression in endometrial cancer and in converting tamoxifen from an estrogen receptor-α (ERα) antagonist into an agonist [18], [19]. SRC-2 overexpression has been linked to metastatic prostate cancer [20]. However, among the three SRCs, SRC-3 has been the most heavily implicated as an oncoprotein. SRC-3 overexpression has been found in multiple types of cancers, including breast [21], pancreatic [22], ovarian [23], gastric [24], prostate [25], and colorectal carcinomas [26]. High SRC-3 levels are associated with breast cancer recurrence [27] and SRC-3 overexpression is associated with tamoxifen and other endocrine therapy resistance in breast cancer patients [27]–[30]. Moreover, SRC-3 is associated with tumor metastasis and recurrence in gastric and liver cancers [24], [31]. It is well known that SRC-3 can drive tumorigenesis by interacting with multiple NRs and other diverse transcription factors to enhance their transcriptional activities, including the ERα [32], androgen receptor [33], progesterone receptor [34], thyroid receptor [35], AP-1, NF-κB, STAT-6, and E2F1 [17]. SRC-3 overexpression also can promote spontaneous tumor initiation and progression in an animal overexpression model system [36]. Together these findings demonstrate that SRC-3 is a key oncoprotein involved in cancer initiation, progression and metastatic growth, pointing to its importance as an important target for therapy [37].

Already, as a proof-of-principle, we characterized the small molecule compounds gossypol and bufalin as SRC small molecule inhibitors (SMIs) [38], [39]. Here, a high-throughput screening assay was performed to identify improved SRC SMIs leading to the identification of verrucarin A as a potent SRC inhibitor that is structurally unrelated to gossypol or bufalin. Verrucarin A inhibits all three SRCs at higher doses, but can selectively reduce SRC-3 protein levels at lower concentrations without impacting CARM-1 or p300 protein levels. Furthermore, verrucarin A showed cytotoxic effects against various types of cancer cells but not normal liver cells, and the potencies for its cytotoxic effects are consistent with those needed to induce SRC-3 protein down regulation. Importantly, we found that verrucarin A does not detectably bind SRC-3 at its effective concentration in cell culture, implicating an upstream effector of SRC-3 as a likely target of this compound.

## Materials and Methods

### Chemicals, reagents and antibodies

Verrucarin A, gemcitabine, docetaxel, tamoxifen, and paclitaxel were obtained from Sigma-Aldrich (St. Louis, MO, USA) and dissolved in DMSO. Gefitinib and BEZ235 were purchased from Selleck Chemicals (Houston, TX, USA). Estradiol (E2) was purchased from Sigma and dissolved in ethanol. SRC-1 and SRC-3 antibodies were purchased from Cell Signaling Technology (Danvers, MA, USA) and CARM1 and SRC-2 antibodies were purchased from Bethyl Laboratories (Montgomery, TX, USA). β-actin and p300 antibodies were obtained from Santa Cruz Biotechnology (Santa Cruz, CA, USA). Lipofectamine 2000 was purchased from Invitrogen (Carlsbad, CA, USA).

### Cell culture

All human cancer cell lines were obtained from the American Type Culture Collection (Manassas, VA, USA), cultured in DMEM (HeLa and MCF-7), RPMI-1640 (A549 and H1299), DMEM/F12 (PC-3), or MEM (HepG2), supplemented with 10% fetal bovine serum (Invitrogen), 100 units/mL penicillin and 100 µg/mL streptomycin (Invitrogen), in a 5% CO_2_ humidified atmosphere at 37°C. Primary mouse hepatocytes were isolated as previously described [39].

### 1,536-well plate SRC-1 and SRC-3 HTS assays

A detailed protocol for the HTS screening assay can be found on the PubChem Bioassay website (http://pubchem.ncbi.nlm.nih.gov/assay/assay.cgi?aid=588357&loc=ea_ras).

### Cell viability assay

Cancer cell lines were plated into 96-well plates at a density of 4×10^3^ cells/100 µL medium per well. After adherence, cells were treated with various concentrations of verrucarin A individually or in combination with other anti-cancer drugs for 72 h, with DMSO vehicle as a control. After treatment, relative numbers of viable cells were measured using the Cell Titer 96 Aqueous One Solution Cell Proliferation Kit (Promega, Madison, WI) according to previous described [40].

### Luciferase reporter assay

Luciferase assays were performed as previously described [41]. For coactivator intrinsic activity assays, the pG5-LUC GAL4-responsive reporter plasmid (Promega) was co-transfected along with mammalian expression vectors encoding full length SRCs fused to the DNA-binding domain of GAL4 (pBIND-SRC-1, pBIND-SRC-2 and pBIND-SRC-3). For ERα/SRC coactivator assays, an estrogen-responsive reporter construct (pERE-E1b-LUC) was cotransfected along with mammalian expression vectors for ERα(pCR3.1-hERα) and SRCs (pCR3.1-SRC-1, pCR3.1-SRC-2 or pCR3.1-SRC-3). Briefly, cells were seeded in a 24-well plate and transfected with expression vectors for ERα, SRC or GAL4 DNA binding domain-SRC fusion proteins along with appropriate luciferase reporter plasmids. Twenty-four hours after transfection, cells were treated with various concentrations of verrucarin A for an additional 24 h. After treatment, cells were lysed and then luciferase activities were measured according to the manufacturer's protocol (Promega).

### Immunoblot analysis

Cell lysates were loaded with equal amounts of protein onto 4–15% SDS–polyacrylamide gels, electrophoresed and transferred to PVDF membranes (Bio-Rad, Hercules, CA, USA). Membranes were blocked for 1 h in TBS-Tween-20 containing 5% non-fat milk and then incubated with primary antibodies at 4°C overnight. After washing, the blots were incubated with horseradish peroxidase (HRP)-linked secondary antibodies (Cell Signaling) at room temperature for 1 h. The blots were visualized with SuperSignal West Pico Chemiluminescent Substrate (Thermo Fisher Scientific, Rockford, IL, USA) according to the manufacturer's instructions.

### Quantitative PCR analysis

Quantitative PCR assay was performed as previously described [42]. Briefly, total RNA was isolated from cells using the RNeasy mini kit (QIAGEN, Valencia, CA, USA). RNA was converted to cDNA with a Reverse Transcription System (Promega) according to the manufacturer's instructions. Quantitative polymerase chain reaction (QPCR) transcript level determination was performed using a SYBR Green PCR Master Mix (Life Technologies, Grand Island, NY, USA) and an ABI Prism 7700 sequence detection system (Life Technologies). The specific primers for QPCR were chosen using the PrimerBank website (http://pga.mgh.harvard.edu/primerbank/index.html) indicated below:

5′- AATGAATACGAGCGTCTACAGC-3′

and 5′- TTTCGTCGTGTTGCCTCTTGA-3′ for SRC-1, 5′- TGGGGCCTATGATGCTTGAG-3′

and 5′- GGTTTTTGACAAATTCCGTGTGG-3′ for SRC-2, 5′- AGACGGGAGCAGGAAAGTAAA-3′

and 5′- GTAAAAGCGGTCCTAAGGAGTC-3′ for SRC-3, 5′- GGAGCGAGATCCCTCCAAAAT-3′

and 5′- GGCTGTTGTCATACTTCTCATGG-3′ for GAPDH.

The specific primers for MMP-2 and MMP-13 QPCR were from previously described [43]:

5′- TGAGCTCCCGGAAAAGATTG-3′

and 5′- TCAGCAGCCTAGCCAGTCG-3′ for MMP-2, 5′- GCAGTCTTTCTTCGGCTTAG-3′

and 5′- CAGGGTCCTTGGAGTGGTCA-3′ for MMP-13.

### Fluorescence spectrometry assay

This assay was performed as previously described [39]. Briefly, the GST fusion proteins of three SRC-3 fragments were expressed and purified. Fluorescence spectrometric assays were performed using an Agilent Cary fluorescence spectrometer (Agilent Technologies Inc., Santa Clara, CA). GST-SRC-3 GST-bHLH, GST-RID, or GST-CID protein was added in a fluorescence cuvette. After addition of verrucarin A or DMSO as a negative control, the protein samples were excited by UV light at a wavelength of 278 nm with a 2-nm bandwidth, and the emission spectrum was recorded from 295 nm to above 400 nm with a bandwidth of 4 nm. The peak fluorescence intensity was 306 nm for GST-bHLH and GST-CID, and 338 nm for GST-RID. Gossypol and bufalin, two published inhibitors of SRC-3, were used as positive controls for this assay.

### Wound healing assay

Cells were grown in 24-well plates to confluence and then a “wound” was created by scratching the cell monolayer using a pipette tip. Cells were treated with verrucarin A for 18 h. Photo documentation was taken at the zero time point and the 18 h time point after wounding for three independent experiments.

### Statistical analysis

The Student's t-test was used to compare the significance of the differences between two groups of data. A value of P<0.05 was regarded as indicating a significant difference.

## Results

### Identification of verrucarin A as a SRC SMI

A high throughput luciferase assay-based screen of a MLPCN chemical library with 359,484 compounds [38], [44] was performed to identify compounds capable of inhibiting the intrinsic transcriptional activities of SRC-3 (PubChem AID:588362), SRC-1 (PubChem AID:588354) and SRC-2 (PubChem AID:651957). Compounds were assayed by measuring luciferase expression from cells transiently transfected with a GAL4 responsive luciferase reporter (pG5-LUC) and an expression vector for either a GAL4 DNA binding (DBD) or GAL4 DBD SRC-1, SRC-2 and SRC-3 fusion proteins. Those compounds that blocked luciferase activity greater than 3σ over DMSO were scored as SMI hits. In our primary screens, the transfected HEK293 cells were treated with test compounds at a concentration of 3.6 µM in 0.36% DMSO for SRC-1 and SRC-3 or 8.9 µM for SRC-2. 3.6 µM gossypol was used as a positive control which was able to elicit 100% inhibition. Based upon a 3σ cut-off, 428 out of 359,498 (0.12%) compounds were able to inhibit SRC-1, 620 out of 359,245 (0.17%) compounds were able to inhibit SRC-2 and 621 out of 359,484 compounds inhibited SRC-3 (0.17%). A summary of SRC inhibitor screening results is shown in Table S1.

**Figure 1:**
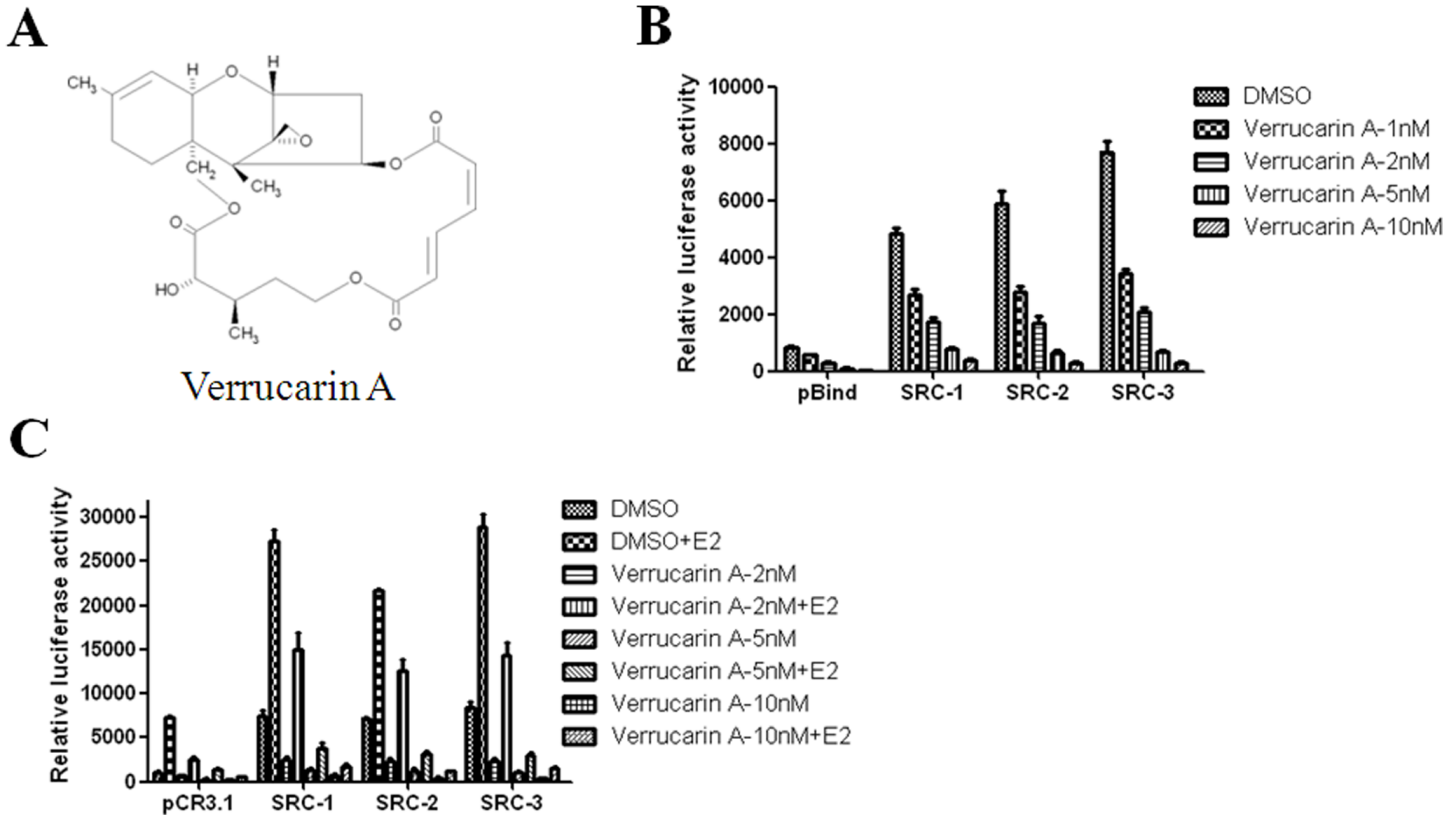
Verrucarin A reduces the transcriptional activities of SRCs in HeLa cells. (A) Chemical structure of verrucarin A. (B) Verrucarin A inhibits pBIND-SRC luciferase activity. HeLa cells were transiently cotransfected with expression vectors for pBIND-SRC-1, pBIND-SRC-2 or pBIND-SRC-3 and the GAL4-responsive pGL5 reporter plasmid before incubation with verrucarin A at different concentrations (0, 1, 2, 5, and 10 nM) for 24 h, followed by luciferase assay. Empty pBIND vector was transfected as a negative control. (C) Verrucarin A inhibits SRC coactivation of ERα. Luciferase assays were performed in HeLa cells transiently transfected with an ERE-luc reporter vector and expression vectors for ERα, and pCR3.1-SRC before incubation with 10 nM E2 and verrucarin A at different concentrations (0, 2, 5, and 10 nM) for 24 h.

Verrucarin A was identified from these screens (Fig. 1A), belonging to a group of sesquiterpene toxins, derived from the pathogenic fungus *Myrothecium verrucaria* often found in infected food grains (Fig. 1A). Verrucarin A has been shown to be cytotoxic at sufficient levels and a variety of mechanisms have been proposed for its biological activity. As one example, it has been shown to inhibit protein synthesis by preventing peptidyl transferase activity [45]. Verrucarin A also can trigger a ROS-mediated intrinsic mechanism of apoptosis [46], and can induce TRAIL-induced apoptosis by eIF2α/CHOP-dependent DR5 induction via ROS generation [47]. Jayasooriya *et al*. showed that verrucarin A enhances TNF-α-induced apoptosis via NF-κB-dependent Fas overexpression [48], [49]. Although, the predominant mechanism of verrucarin A-induced cell growth inhibition remains unclear, it and its derivatives are considered to be potentially useful anticancer agents [46], [48], [50].

To confirm the ability of verrucarin A to inhibit SRCs as seen in our primary screens, HeLa cells were transiently cotransfected with expression vectors for GAL4-DBD-SRCs (pBIND-SRCs) and a GAL4-responsive luciferase reporter (pGL5) plasmid before incubation with verrucarin A at various concentrations (0, 1, 2, 5, and 10 nM) for 24 h. Verrucarin A inhibited GAL4-responsive luciferase reporter activity of all SRCs in a dose-dependent manner (Fig. 1B). Furthermore, to evaluate SRC coactivator activities on a NR, HeLa cells were transfected with an estrogen response element (ERE) containing reporter gene and expression vectors for ERα, and SRC-1, SRC-2 and SRC-3. Twenty-four hours after transfection, cells were incubated with 10 nM E2 and verrucarin A at the indicated concentrations (0, 2, 5, and 10 nM) for 24 h followed by luciferase assays. These results indicate that verrucarin A can block SRC mediated coactivation of ERα consistent with its ability to inhibit coactivator intrinsic transcriptional activity (Fig. 1C).

### Verrucarin A selectively reduces SRC-3 protein levels

**Figure 2:**
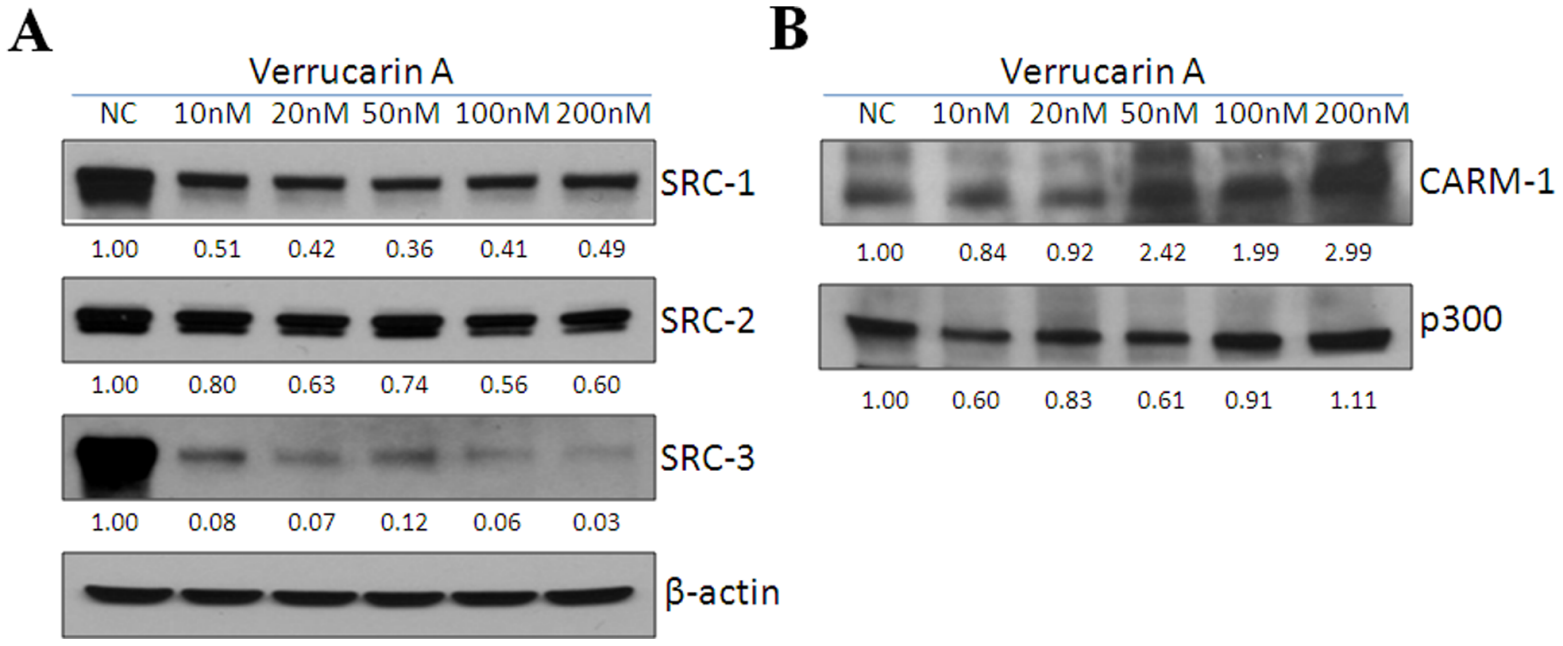
Verrucarin A selectively reduces SRC-3 protein levels while it does not reduce CARM-1 and p300 protein levels. (A-B) A549 cells were treated with verrucarin A at different concentrations (0, 10, 20, 50, 100, and 200 nM) for 24 h, then Western analysis was performed to quantitate SRC-1, SRC-2, SRC-3, CARM1, and p300 proteins.

Since verrucarin A inhibits the transcriptional activities of all three SRCs, we wished to investigate if this was due to downregulation of protein expression of SRCs as we have observed for gossypol and bufalin [38], [39]. A549 cells were treated with verrucarin A at various concentrations (0, 10, 20, 50, 100, and 200 nM) for 24 h, then protein levels for SRC-1, SRC-2, and SRC-3 were determined by Western analysis. Verrucarin A treatment was able to reduce SRC-3 protein expression by 90% at 10 nM, but reduced SRC-2 and SRC-1 to a smaller extent and only at much higher doses (Fig. 2A). Moreover, verrucarin A treatment did not reduce protein levels of CARM-1 and p300 (Fig. 2B). Verrucarin A also reduced SRC-3 protein levels in other cancer cells, such as LNCaP, PC-3, and MCF-7 cell lines (Fig. S1). These data suggest that verrucarin A can selectively reduce levels of SRC-3 at low nanomolar concentrations.

### Verrucarin A is selectively cytotoxic to cancer cells

**Figure 3:**
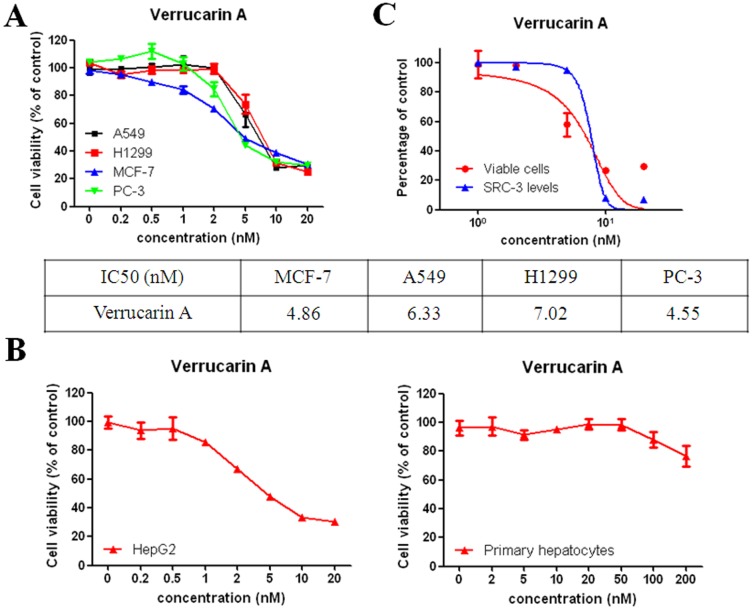
Verrucarin A can selectively kill cancer, but not normal cells. (A) Verrucarin A can kill a variety of cancer cells. MCF-7, A549, H1299, and PC-3 cells were treated with verrucarin A at different concentrations (0, 0.2, 0.5, 1, 2, 5, 10, and 20 nM) for 72 h, followed by MTS assay. (B) HepG2 cells are sensitive to verrucarin A, but primary hepatocytes are not. HepG2 cells were treated with verrucarin A at different concentrations (0, 0.2, 0.5, 1, 2, 5, 10, and 20 nM) for 48 h. Primary hepatocytes were treated with verrucarin A at different concentrations (0, 2, 5, 10, 20, 50, 100, and 200 nM) for 48 h, followed by MTS assay. (C) Verrucarin A inhibits cancer cell viabilities with potencies in line with its ability to down regulate SRC-3 protein levels.

To test if verrucarin A-mediated downregulation of SRC-3 corresponds with cell growth inhibition, MCF-7, A549, H1299, and PC-3 cancer cells were treated with verrucarin A at different concentrations (0, 0.2, 0.5, 1, 2, 5, 10, and 20 nM) for 72 h and cell growth was determined by MTS assay. All four of these cell lines were sensitive to verrucarin A, with IC_50_ values ranging from 4 to 8 nM (Fig. 3A). To investigate whether verrucarin A has cytotoxic effects on non-immortalized, non-transformed cells, we tested the cytotoxic effects of verrucarin A on hepatocellular HepG2 carcinoma cells and compared them against mouse primary hepatocytes. HepG2 cells and mouse primary hepatocytes were treated with verrucarin A at different concentrations for 48 h, followed by MTS assays. We found that like other cancer cell lines, HepG2 cells were sensitive to verrucarin A, with an IC_50_ value of 4.90 nM. In contrast, mouse primary hepatocytes still can survive even with verrucarin A concentrations as high as a 200 nM (Fig. 3B). Moreover, A549 cells were treated with verrucarin A at different concentrations (0, 1, 2, 5, 10, and 20 nM) for 72 h, followed by determination of SRC-3 protein levels (Fig. S2). Compared with the results of verrucarin A inhibiting A549 cell growth, verrucarin A inhibits cancer cell viability with potencies in line with its ability to downregulate SRC-3 protein levels (Fig. 3C).

### Verrucarin A inhibits SRC-3 downstream target gene (MMP-2 and MMP-13) expression

**Figure 4:**
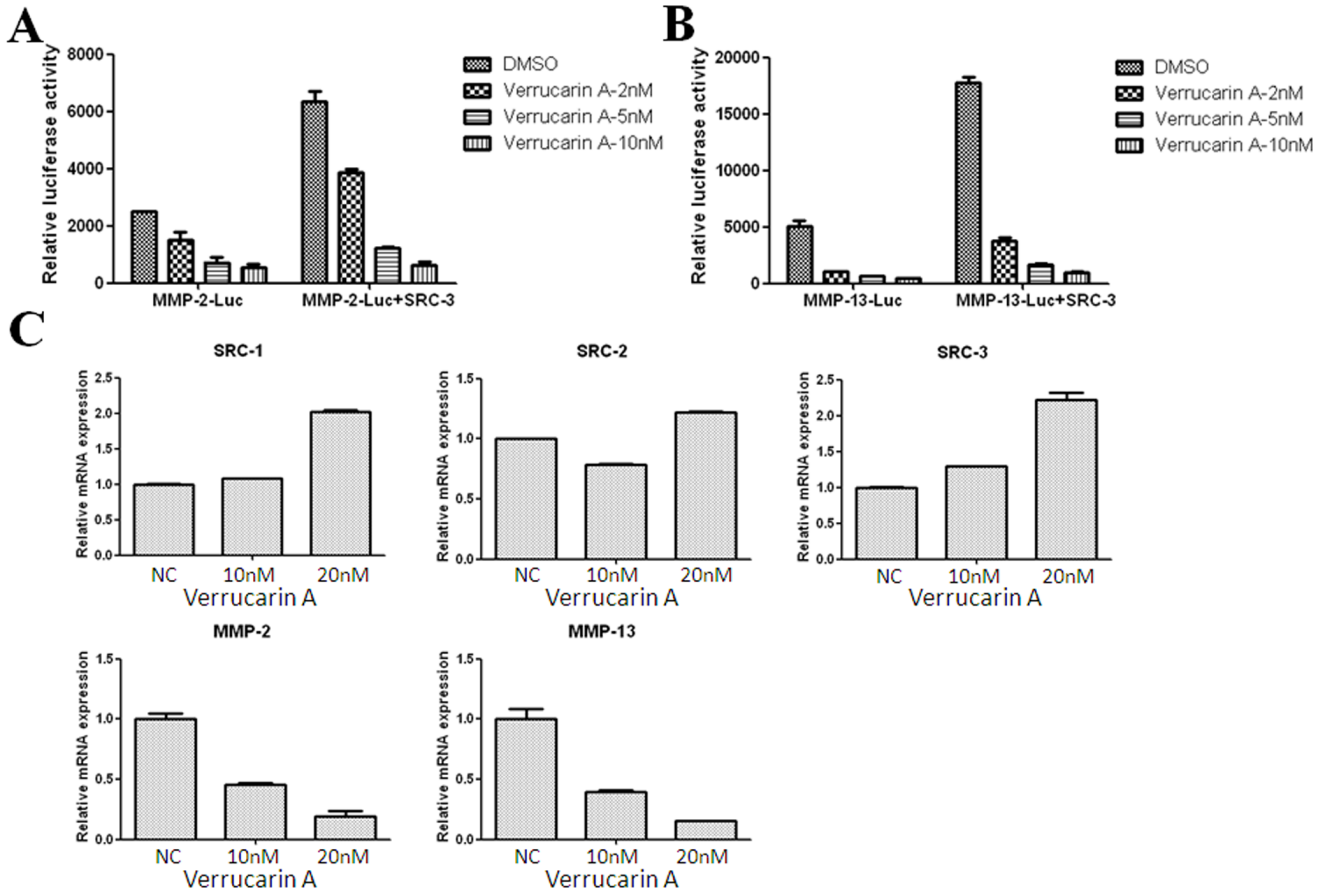
Verrucarin A inhibits SRC-3 downstream target gene (MMP2 and MMP13) expression. (A–B) Luciferase assays were performed in HeLa cells transiently transfected with MMP2-Luc, MMP13-Luc, and pCR3.1-SRC-3 expression vectors before incubation with verrucarin A at different concentrations (0, 2, 5, and 10 nM) for 24 h. (C) H1299 cells were treated with verrucarin A at different concentrations (0, 10, and 20 nM) for 24 h, then real-time PCR was performed to quantitate SRC-1, SRC-2, SRC-3, MMP2, and MMP13 mRNA expression.

Above, we demonstrated that verrucarin A reduces cellular SRC-3 protein. To investigate if verrucarin A regulates SRC-3 downstream target gene expression (MMP2 and MMP13), HeLa cells were transiently transfected with MMP2-Luc or MMP13-Luc reporter constructs and an SRC-3 expression vector before incubation with verrucarin A at different concentrations (0, 2, 5, and 10 nM) for 24 h, followed by luciferase assays [43]. Verrucarin A reduced luciferase expression driven from the MMP2 and MMP13 reporter genes (Fig. 4A). To assess the impact on endogenous MMP2 and MMP13 genes, H1299 cells were treated with verrucarin A for 24 h, then real-time PCR was performed to assess SRC-1, SRC-2, SRC-3, MMP2, and MMP13 mRNA expression. Verrucarin A inhibited MMP2 and MMP13 mRNA expression, consistent with what was observed in the reporter gene assays (Fig. 4B). Notably, the mRNA levels of for each of the three SRCs (Fig. 4C) were not downregulated, suggesting that verrucarin A reduces SRC-3 protein levels through a posttranscriptional mechanism. To explore the kinetics of verrucarin A on promoting SRC-3 protein downregulation, a time-course analysis was performed. A549 cells were treated with 20 nM verrucarin A at a series of time points (0, 0.5, 1, 2, 4, and 6 h) and then SRC-3 protein levels were examined by Western analysis. As shown in Fig. S3, a decrease in SRC-3 protein was observed 2 h after administration of verrucarin A.

### Verrucarin A inhibits H1299 cell migration

**Figure 5:**
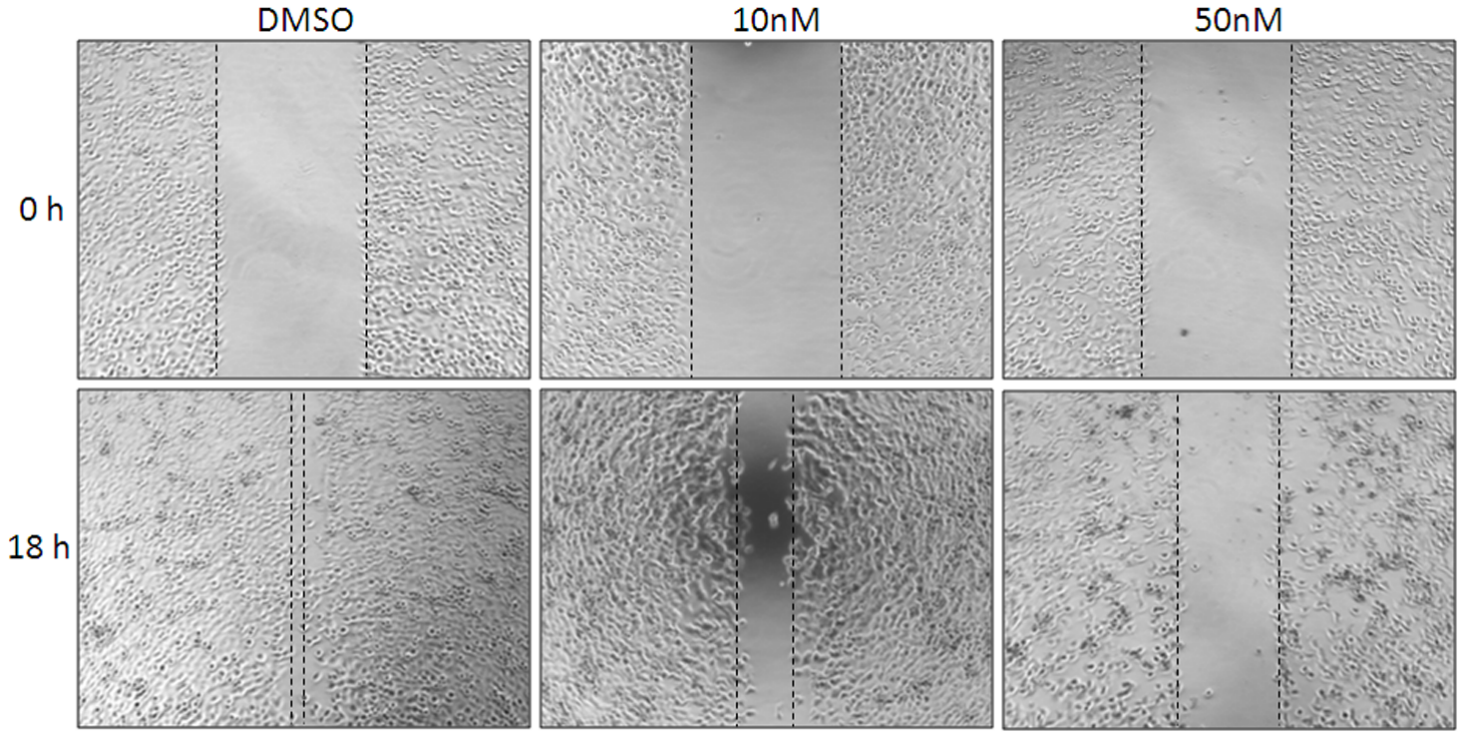
Verrucarin A inhibits H1299 cell migration. H1299 cells were plated into 24-well plates and treated with verrucarin A for 18 h for wound healing assay analysis.

Previous studies have demonstrated that SRC-3 plays important roles in tumor initiation and expansion, and functions as a critical coactivator that drives tumor cell invasion and metastasis [43], [51], [52]. To evaluate whether verrucarin A can block cell motility concomitantly with inhibition of SRC-3, H1299 cells were plated into 24-well plates and treated with verrucarin A for 18 h and cell motility was determined using a wound healing assay (see Materials and Methods). As shown in Fig. 5, H1299 cell migration was attenuated by verrucarin A.

### Verrucarin A's SMI mechanism of action does not involve direct binding to SRC-3

Next, we sought to determine if verrucarin A physically interacts with SRC-3. We examined the ability of verrucarin A to quench the intrinsic fluorescence of different portions of the SRC-3 protein. The fluorescence emission maximum of glutathione S-transferase (GST) SRC-3 RID at 330 nm was not quenched by verrucarin A at concentrations below 10 mM which is well beyond its effective concentration in cell culture (data not shown). The ability of verrucarin A to quench the fluorescence of the GST SRC-3 CBP interaction domain (CID) and basic helix-loop-helix (bHLH) constructs were similarly evaluated and no nanomolar affinity binding was observed (data not shown). These data point to the likelihood that verrucarin A does not inhibit SRC-3 by directly binding to SRC-3, but instead influences the function of an upstream mediator of SRC-3 function and protein stability.

### Verrucarin A increases cancer cell chemosensitivity to other anti-cancer drugs

**Figure 6:**
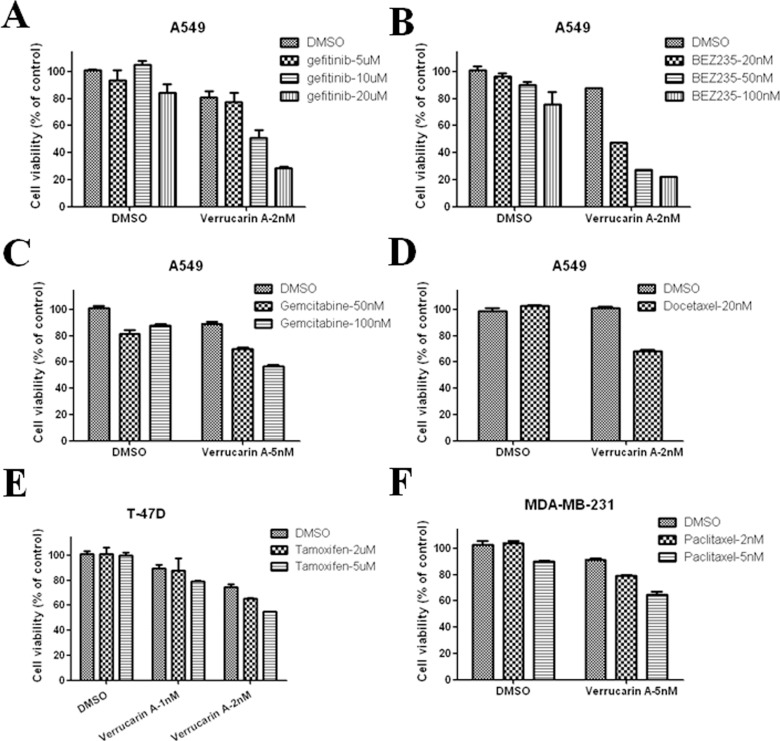
Verrucarin A increases cancer cell chemosensitivity to other anti-cancer drugs. (A–D) A549 cells were treated with verrucarin A in combination with gefitinib, BEZ235, gemcitabine, or docetaxel. (E) T-47D cells were treated with verrucarin A in combination with tamoxifen. (F) MDA-MB-231 cells were treated with verrucarin A in combination with paclitaxel. All cells were treated for 72 h, followed by MTS assay.

Numerous studies have revealed that SRC-3 is an integrator of growth promoting signaling pathways including the EGFR, HER2 and NR signaling pathways and that SRC-3 contributes to chemoresistance when it is overexpressed [30], [53]. Therefore, it is expected that inhibition of SRC-3 should sensitize cancer cells to anti-cancer drugs by simultaneously blocking multiple growth signaling pathways. First, we examined the inhibitory effects of verrucarin A on cell viability in combination with four widely-used chemotherapeutic drugs (docetaxel, gemcitabine, BEZ235, and gefitinib) to block cell growth in A549 lung cancer cells. Treating A549 cells with gefitinib alone at concentrations ranging from 5 µM to 20 µM, BEZ235 from 20 nM to 100 nM, gemcitabine of 50 nM and 100 nM, or docetaxel at 20 nM had no observable effects on cell viability. However, when combined with 2 nM or 5 nM verrucarin A, the four anti-cancer drugs all inhibited cell growth in a dose-dependent manner (Fig. 6A–D). Next, we evaluated the synergistic effects of paclitaxel and tamoxifen in combination with verrucarin A on breast cancer cell viability. Exposure to a low concentration of verrucarin A significantly sensitized T-47D and MDA-MB-231 cells to tamoxifen and paclitaxel, respectively (Fig. 6E and F). These results are consistent with SRC-3′s role as an integrator of multiple growth factor signaling cascades and that its partial inhibition with verrucarin A can effectively increase cancer cell chemosensitivity to other anti-cancer drugs.

## Discussion

SRC-3 overexpression has been demonstrated in a wide range of cancers as discussed above. As a coactivator for NRs and many other transcription factors, SRC-3 simultaneously drives the activity of multiple cellular signal transduction pathways. Currently, most targeted cancer drugs typically only block a single target or signaling pathway, and their clinical efficacy is frequently limited. Cancer cells typically acquire resistance to individual anticancer agents by the activation of alternative, escape growth pathways. For example, the PI3K-Akt pathways are commonly activated in ER positive breast cancer cells and can promote cell growth and confer resistance to tamoxifen [54]. However, the response of breast cancers to chronic PI3K-Akt inhibition is often limited [55], suggesting that additional growth factor escape pathways are activated. SRC-3 is a central integrator of multiple steroid hormone and hormone-independent signal transduction pathways, including the IGF-1/Akt [56], [57], NF-κB [58], EGFR [59], E2F1 [60], [61], and MAPK signal pathways [62], [63]. Therefore, SMIs that can disrupt SRC-3 function should simultaneously prevent the activation of such a large breadth of growth pathways that underlie critical steps in cancer initiation, expansion, metastasis, and chemoresistance, that the cancer cell would be less able to overcome resistance to a SRC-3 SMI.

While the characterization of gossypol as a SRC SMI [39] validated the concept that SRCs could be targeted with a SMI, limitations in its potency provided the impetus for us to pursue high throughput screening to identify improved SRC SMIs, this ultimately led to the identification of improved SMIs such as bufalin [38] and verrucarin A. We showed that gossypol can reduce cellular protein concentrations of SRC-1 and SRC-3, without altering protein expression of SRC-2, or other coactivators, such as p300 and CARM1 [39] but only at concentrations of 5 µM which are not achievable *in vivo*. In this study, we found that verrucarin A can degrade 90% of SRC-3 protein expression at a 10 nM concentration, and about 50% of SRC-1 and SRC-2 proteins at 200 nM (Fig. 2A). Additionally, verrucarin A did not downregulate protein levels of other coactivators such as CARM-1 and p300 (Fig. 2B). Importantly, chemotherapeutic agents should be able to selectively kill tumor cells while not affecting normal cells and we found that it kills hepatocellular carcinoma HepG2 cells but not primary hepatocytes (Fig. 3B). Based on these findings, verrucarin A has promising selectivity toward tumor cells, pointing to itself and its derivatives as candidate SMIs for further development as anti-cancer agents.

In conclusion, we have identified verrucarin A as a potent, SRC-3-selecitve SMI. Clinical experience has demonstrated that combination chemotherapy regimens designed to target two distinct tumor growth pathways are often more effective than monotherapy, but even in these instances, poor response is often observed. We posit that this is due to the existence of a breadth of additional growth factor pathways available to the cancer cell. Because SRC-3 coactivates such a broad number of signaling pathways involved in cancer initiation, proliferation, motility and invasion, it stands out as a novel but promising target to combat chemoresistance. We also show that consistent with this idea, verrucarin A was able to sensitize cancer cells to a variety of established cancer drugs, highlighting the potential for SRC-3 as a key target for advanced, therapy-resistant cancers.

## Supporting Information

Figure S1 **Verrucarin A inhibits SRC-3 protein expression, but does not significantly reduce CARM1 and p300 protein expression in PC-3, LNCaP, and MCF-7 cells.** Cells were treated with the indicated concentrations of verrucarin A for 24 h.
(TIF)

Figure S2 **Verrucarin A downregulates SRC-3 protein expression in A549 cells.** Cells were treated with the indicated concentrations of verrucarin A for 72 h and cell lysates were analyzed by Western blotting.
(TIF)

Figure S3 **Verrucarin A induces SRC-3 protein degradation in lung cancer cells.** A549 cells were treated with 20 nM verrucarin A at the indicated time points (0, 0.5, 1, 2, 4, and 6 h), and then SRC-3 protein levels were examined by Western analysis.
(TIF)

Table S1 **Summary of SRC inhibitor screening.**
(XLSX)
