# Supplementary figures and images for: Identification of Verrucarin A as a Potent and Selective Steroid Receptor Coactivator-3 Small Molecule Inhibitor
Source: PLoS One. 2014 Apr 17;9(4):e95243. doi: 10.1371/journal.pone.0095243 (PMC3990629; doi:10.1371/journal.pone.0095243)

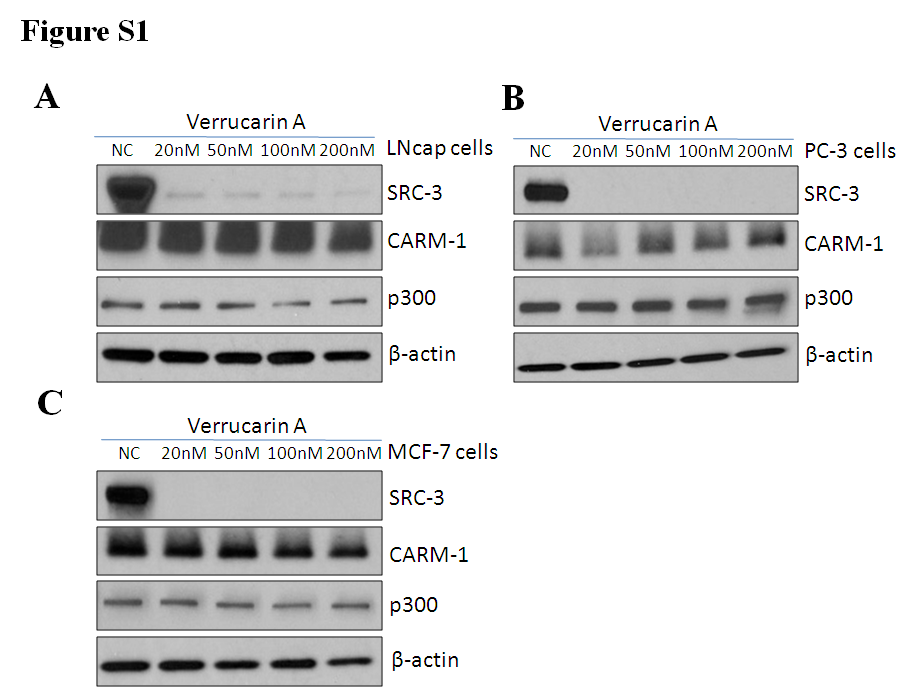

Supplement: Figure S1 — Verrucarin A inhibits SRC-3 protein expression, but does not significantly reduce CARM1 and p300 protein expression in PC-3, LNCaP, and MCF-7 cells. Cells were treated with the indicated concentrations of verrucarin A for 24 h. (TIF) [file pone.0095243.s001.tif]

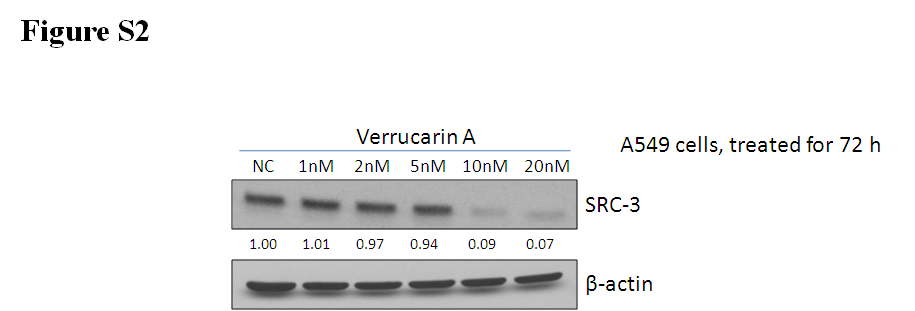

Supplement: Figure S2 — Verrucarin A downregulates SRC-3 protein expression in A549 cells. Cells were treated with the indicated concentrations of verrucarin A for 72 h and cell lysates were analyzed by Western blotting. (TIF) [file pone.0095243.s002.tif]

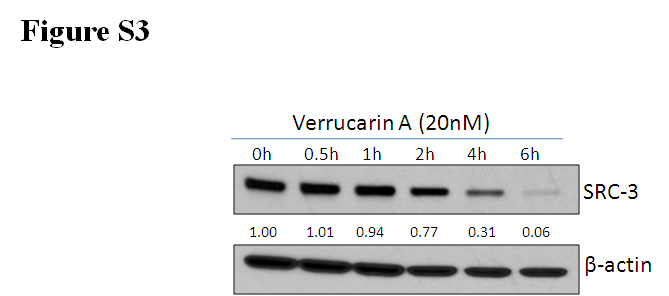

Supplement: Figure S3 — Verrucarin A induces SRC-3 protein degradation in lung cancer cells. A549 cells were treated with 20 nM verrucarin A at the indicated time points (0, 0.5, 1, 2, 4, and 6 h), and then SRC-3 protein levels were examined by Western analysis. (TIF) [file pone.0095243.s003.tif]
